# Conduction system pacing versus biventricular pacing in heart failure with reduced ejection fraction and electrical dyssynchrony

**DOI:** 10.3389/fcvm.2024.1495689

**Published:** 2024-12-05

**Authors:** Ahmed Ammar, Ahmed Elewa, Amr Y. Emam, Mohamed Sharief, Omnia Kamel

**Affiliations:** ^1^ Cardiology Department, Worcestershire Acute Hospitals NHS Trust, Worcester, United Kingdom; ^2^ Cardiology Department, Ain Shams University, Cairo, Egypt; ^3^ Cardiology Department, Aswan Heart Center, Aswan, Egypt; ^4^ Internal Medicine, Mersey and West Lancashire Teaching Hospitals NHS Foundation Trust, Liverpool, United Kingdom

**Keywords:** conduction system pacing, His bundle pacing, left bundle branch area pacing, left bundle branch pacing, biventricular pacing, heart failure with reduced ejection fraction, electrical dyssynchrony

## Abstract

Biventricular pacing (BiVP) has been the cornerstone of cardiac resynchronization therapy (CRT) in the management of symptomatic heart failure patients with reduced ejection fraction (HFrEF) and electrical dyssynchrony despite guideline-directed medical therapy (GDMT). However, BiVP has some limitations, including technical difficulties and high non-response rates. Conduction system pacing encompassing His bundle pacing (HBP) and left bundle branch area pacing (LBBAP) has recently emerged as a promising alternative to CRT in this group of patients. In this review, we explore the current evidence, guidelines, limitations, gaps in knowledge, and ongoing trials comparing CSP and BiVP for the management of HFrEF with electrical dyssynchrony.

## Introduction

Biventricular pacing has been the standard device therapy for patients with HFrEF who exhibit electrical dyssynchrony with left ventricular ejection fraction (LVEF) ≤35% and QRS duration (QRSd) ≥130 msec especially those with left bundle branch block (LBBB) despite being on maximum tolerated GDMT resulting in marked improvement in symptoms and reduction in morbidity and mortality rates (1, 2). Nonetheless, there are some challenges related to CRT, including high non-responder rates and technical limitations such as the absence of suitable targets, difficulties with lead placement, and phrenic nerve stimulation (3).

Conduction system pacing (CSP), comprising HBP and LBBAP, has recently emerged as a promising alternative to CRT. CSP offers the potential to achieve more physiologic pacing by directly stimulating the His–Purkinje system, thereby restoring synchronous ventricular contraction. Furthermore, it has been found to be a safe, feasible, and effective pacing modality in achieving significant heart function improvement and better clinical outcomes in CRT non-responders, making it a reasonable and promising pacing strategy in this population (4).

## Limitations of BiVP in HFrEF and electrical dyssynchrony

While BiVP has been the standard of care for device therapy for patients with an indication for CRT and electrical dyssynchrony, coronary sinus lead implantation can be associated with technical challenges, including difficulty in coronary sinus cannulation due to abnormal coronary sinus anatomy, inadvertent coronary sinus perforation, phrenic nerve stimulation, absence of good targets, high pacing thresholds due to LV scarring, displacement of the lead, and/or long procedure times resulting in increased risk of infections (3).

Furthermore, 30%–45% of CRT candidates are considered non-responders despite BiVP and do not benefit clinically (5). Patients with permanent atrial fibrillation (AF) might also not benefit from BiVP, and the evidence regarding the efficacy of BiVP-CRT in this subgroup of patients is still very limited (6).

## Current evidence for CSP in HFrEF and electrical dyssynchrony

CSP has recently emerged as a potential alternative for achieving cardiac resynchronization. HBP has demonstrated efficacy in observational and small randomized studies, showing similar clinical and echocardiographic improvements compared to BiVP (7, 8). Furthermore, Huang et al. were the first to report a successful left bundle branch pacing (LBBP) implantation in a heart failure patient with LBBB as a rescue pacing option to HBP. Clinical outcomes after a 1-year follow-up revealed significant improvements, with LVEF increasing from 32% to 62% and left ventricular end-diastolic dimension decreasing from 76 mm to 42 mm. (9).

Vinther and his team in Copenhagen conducted the first randomized trial, known as the His-alternative study, comparing HBP and BiVP in symptomatic heart failure patients with LBBB, with 50 patients randomized to each group. At the 6-month follow-up, the per-protocol analysis revealed significantly higher LVEF and lower left ventricular end-systolic volume in the HBP group compared to the BiVP group. However, the intention-to-treat analysis showed no superior effect between the two groups in terms of QRS duration (7).

The His–Purkinje Cnduction System Pacing Optimized Trial of Cardiac Resynchronization Therapy (HOT-CRT) conducted by Vijayaraman and his team compared the feasibility and clinical efficacy of CSP versus BVP in patients with HFrEF and indication for CRT. CSP using HBP or LBBP was found to be safe, feasible, and associated with greater improvement in LVEF compared with BiVP in patients requiring CRT (10).

The Left Bundle Branch-Optimized Cardiac Resynchronization Therapy (LOT-CRT) trial showed that LBBAP combined with coronary venous left ventricular pacing is feasible and safe and provides greater electrical resynchronization as compared with BiVP only and could be an alternative to BiVP, especially in cases with suboptimal electrical resynchronization (11).

Jastrzębski and his colleagues also showed in their CSPOT study that in patients with CRT indications and advanced conduction disease, LOT-CRT and BVP provided greater acute hemodynamic than LBBAP only and that LOT-CRT reduced QRS duration more than LBBAP or conventional BiVP alone suggesting that patients with wide QRS (QRS ≥171 ms) or deep septal pacing are more likely to benefit from the addition of a left ventricular coronary vein lead to implement LOT-CRT (12).

Feng and his team also showed that LBB-optimized LV pacing (LOT-aCRT) using LBBAP combined with coronary venous pacing was clinically feasible in patients with HFrEF, LBBB, and preserved AV conduction and was associated with significant reduction in QRS duration and improvement in LV function. However, it was associated with longer procedure duration and fluoroscopy time (13).

The Left Bundle Branch Pacing Versus Biventricular Pacing for Cardiac Resynchronization Therapy (LBBP-RESYNC) is another randomized controlled trial (RCT) conducted by Wang and his team which compared LBBP and BiVP among HF patients with non-ischemic cardiomyopathy and LBBB showing that LBBP resulted in greater improvement in LVEF with a mean difference of 5.6% compared with BiVP and reduction in indexed LV systolic volume from baseline to 6 months of follow-up which suggested that LBBP could be a promising first-line resynchronization strategy alongside BiVP-CRT in clinical practice for patients with non-ischemic cardiomyopathy and LBBB (14).

In the observational multi-center study conducted by Diaz and his colleagues in 2023, LBBAP as an initial CRT strategy resulted in a lower risk of HF-related hospitalizations, a reduction in procedural and fluoroscopy times, shorter paced QRSd, and improvements in LVEF compared with BiVP among 371 patients (15).

Vijayaraman and his colleagues also showed that LBBAP improved clinical outcomes compared with BiVP among 1,778 patients with CRT indications and that LBBAP may be a reasonable alternative to BiVP with greater improvement of LVEF and the composite endpoint of time to death or heart failure hospitalization (16).

Chen and his colleagues in 2021 also found that LBBP-CRT is associated with better electromechanical resynchronization compared to optimized BiVP with an adaptive algorithm with a significant reduction in QRSd in patients with LBBB, LVEF ≤35%, and HF over 12 month follow-up period. Furthermore, LBBP-CRT demonstrated higher clinical and echocardiographic response, especially higher super-response (≥20% absolute increase or LVEF ≥50%) compared to BiVP-CRT with an adaptive algorithm (17).

In 2024, Shroff and his colleagues demonstrated that LBBAP-CRT is at least as effective as BiVP-CRT in appropriately selected patients with HF. In a study conducted on 479 consecutive patients referred with heart failure, LBBAP-CRT was associated with early recovery in LVEF because of its prominent resynchronizing effect on the LV through recruitment of the native conduction system, resulting in significant gains in functional status and QoL improvement over BiVP-CRT in addition to improved lead performance with LBBAP-CRT (18).

A meta-analysis of four non-randomized controlled trials including 249 patients and comparing LBBAP versus BiVP-CRT also confirmed the significantly shortened QRSd, improved LVEF and NYHA class, and better echocardiographic and clinical response rate with LBBAP (19).

The randomized non-inferiority study, Left Ventricular Activation Time Shortening with Conduction System Pacing vs. Biventricular Resynchronization Therapy (LEVEL-AT), led by Pujol Lopez and her colleagues, compared the efficacy of CSP versus BiVP in achieving ventricular resynchronization in symptomatic patients with HFrEF who were on optimal medical therapy and met the criteria for cardiac resynchronization therapy (CRT). The trial enrolled 35 patients in each group, with LBBP attempted in the CSP group for 28 patients, achieving an 82% implantation success rate. After six months of follow-up, the study found comparable outcomes between CSP and BiVP in terms of cardiac resynchronization, ventricular reverse remodeling, and clinical improvement (20).

Moreover, the I-CLAS study published by Vijayaraman and his colleagues in 2024 showed that LBBAP was associated with a significantly lower incidence of new-onset AF and sustained ventricular tachycardia (VT) or ventricular fibrillation (VF) compared with BiVP in patients undergoing CRT suggesting that physiological resynchronization using LBBAP may lower the incidence of atrial and ventricular arrhythmias compared with BiVP (21).

Finally, the result of Conduction System Pacing vs. Biventricular Resynchronization Therapy in Systolic Dysfunction and Wide QRS (CONSYST-CRT) randomized trial comparing CSP vs. BiVP in 134 patients with indications for CRT was recently presented in the European Society of Cardiology (ESC) annual conference in London 2024 showing non-inferiority of CSP (mainly LBBAP) versus BiVP achieving similar clinical and echocardiographic outcomes (22).

The current evidence for CSP in HFrEF and electrical dyssynchrony has been summarised in Table 1.

**Table 1:** Summary of published studies on CSP versus BiVP in patients with indication for CRT and electrical dyssynchrony.

Study	Year	Design	Comparison	Number of patients	Outcomes
Chen et al. (17)	2021	Observational, multi-center	LBBP vs. BiVP with an adaptive algorithm	100	LBBP resulted in a more significant reduction in QRS duration and a higher super-response rate compared to BiVP
His-alternative study, Vinther et al. (7)	2021	Randomized, single center	HBP vs. BiVP	50	At 6 months of follow-up, per-protocol LVEF was significantly higher in the HBP group with a lower LV end-systolic volume compared to the BiVP group
Feng et al. (13)	2022	Observational, single center	LBBAP and LV pacing (LOT-aCRT) vs. BiVP	21	LOT-aCRT was associated with significant narrowing of the QRSd and improvement in LV function vs. BiVP
LBPP-RESYNC, Wang et al. (14)	2022	Randomized, multi-center	LBBAP vs. BiVP	40	LBBAP demonstrated greater LVEF improvement with greater reductions in left ventricular end-systolic volume and NT-proBNP as compared with BiVP-CRT in heart failure patients with non-ischemic cardiomyopathy and LBBB
LEVEL-AT, Lopez et al. (4)	2022	Randomized, single center	CSP vs. BiVP	70	CSP was non-inferior to BiVP in resynchronization, ventricular reverse remodeling, and clinical outcomes
LOT-CRT, Jastrzębski et al. (11)	2022	Observational, multi-center	(LOT-CRT) vs. BiVP		LOT-CRT was feasible and safe and provides greater electrical resynchronization with greater narrowing of QRS complex as compared to BiVP
HOT-CRT, Vijayaraman et al. (10)	2023	Randomized, multi-center	HBP vs.. BiVP	160	HBP resulted in significantly greater improvement in LVEF as compared to BiVP with similar improvements in QRSd, LV end-systolic volumes, and QOL
Diaz et al. (15)	2023	Observational multi-center	LBBAP vs.. BiVP	371	LBBAP was associated with a lower risk of HF hospitalization and shorter procedure times than as compared to BiVP
Vijayaraman et al. (16)	2023	Observational, multi-center	LBBAP vs. BiVP	1,778	LBBAP was associated with a significant reduction in the composite outcome of all-cause mortality or HFH, narrowing of QRSd, and improvement in LVEF compared with BVP
I-CLAS, Vijayaraman et al. (21)	2024	Observational, multi-center	LBBAP vs. BiVP	1,414	LBBAP was associated with a lower incidence of new-onset AF, sustained VT, or VF as compared to BiVP
Shroff et al. (18)	2024	Observational, multi-center	LBBAP vs. BiVP	479	LBBAP group showed greater improvement in LVEF at 6 and 12 months accompanied by greater reduction in left ventricular end-systolic volume, QRS duration, and improvement in NYHA class and QoL
CONSYST-CRT, Lopez et al. (22)	2024	Randomized, single center	CSP vs. BiVP	134	CSP was non-inferior to BiVP in achieving all-cause mortality, ≥15% LVESV decrease, NYHA class, and QRS narrowing
CSPOT, Jastrzębski et al. (12)	2024	Observational, multi-center	LBBAP vs. BiVP vs. LOT-CRT	48	LOT-CRT and BiVP alone provided greater acute hemodynamic benefit, with more reduction in QRSd in the LOT-CRT arm

## Comparison between different types of CSP

There is a relative paucity of data about HBP versus LBBAP for CRT, and still, large randomized controlled trials are needed to determine which type of CSP carries the best outcomes in the short and long term. Ali et al. conducted a single-center study comparing the BiVP-CRT versus HBP-CRT and LBBAP-CRT to assess the electrical response via non-invasive mapping and acute hemodynamic response. Both LBBAP and HBP were superior to BiVP-CRT regarding the hemodynamic effect with no marked difference between them. As for the electrical response, LBBAP-CRT produced equivalent LV synchrony, but less total ventricular synchrony compared to HBP-CRT, both being better than BiVP-CRT. This provides a notion that LV synchrony is the main driver for improved hemodynamic response to CRT and that taking into consideration the technical advantages of LBBAP, LBBAP-CRT could be the standard modality for CRT in the future (23).

Myocardial work (MW) has emerged as highly effective tool for quantitatively assessing cardiac mechanical synchrony and efficiency, surpassing speckle-tracking imaging. Furthermore, MW offers a more precise evaluation of synchronization compared to electrocardiographic measures such as QRSd (24). A comparative study on MW performance during spontaneous rhythm, conducted by Azzolini and his colleagues in 2023, found no significant differences in the myocardial work index (MWI) between HBP and LBBAP (25).

In a 2024 retrospective study, Chen and his colleagues found that both LVSP and LBBP yielded similar improvements in LVEF and similar rates of heart failure hospitalization in HFrEF patients undergoing LBBAP for CRT. On the other hand, deep septal pacing (DSP) had no significantly favorable effects on reverse remodeling including LVEF recovery. Additionally, a unipolar tip-paced EGM with a terminal R wave in V1 was an independent predictor in the univariate analysis of good response to CRT (26).

Vijayaraman et al. also found no significant difference in the primary outcome of death or heart failure hospitalization (HFH) in their study comparing clinical outcomes between HBP and LBBAP, where approximately 30% of the patients had an LV ejection fraction of <50%. However, this was an observational non-randomized study that included patients who mainly had an indication for pacing not for CRT (27).

As for the learning curve, using procedural time and fluoroscopy time as surrogates for that parameter, LBBAP was found to have a shorter learning curve (starting to plateau after the first ten cases) compared to HBP where the learning curve started to plateau after 30 cases (28).

A summary outlining the differences among various types of CSP is presented in Table 2.

**Table 2:** Comparison between different types of CSP based on current evidence.

	His bundle pacing (HBP)	Left bundle branch pacing (LBBP)	Left ventricular septal pacing (LVSP)
Hemodynamic response	Superior to BiVP; similar to LBBAP in improving hemodynamics	Comparable to HBP; effective in LV synchronization, potentially becoming standard for CRT	Comparable outcomes in LVEF recovery and HF hospitalization rates, especially in CRT settings
Electrical synchrony	Better total ventricular synchrony than LBBP and LVSP	Equivalent LV synchrony but less total ventricular synchrony compared to HBP	Produces less effective electrical synchrony and reverse remodeling than both HBP and LBBP
Myocardial work index (MWI)	No significant differences compared to LBBP in myocardial work	Comparable to HBP in mechanical performance; efficient synchronization	Not directly assessed but implied to be less effective than HBP/LBBP
Technical challenges	Difficult His engagement, variable capture thresholds, low sensing amplitude	Easier implantation, higher sensing, lower capture thresholds	No specific challenges challengers but high thresholds may be encountered
Learning curve	Steeper; starts to plateau after 30 cases	Shorter; plateaus after the first 10 cases	Shorter than HBP
Procedural time	Longer due to complexity locating the His and achieving a stable position with an acceptable threshold	Shorter procedural time and fewer lead-related complications	Generally comparable to LBBP in procedural simplicity
Lead extractability	High success rate (>95%); mostly removed by simple traction	Similar feasibility of extraction with few complications reported	Less data available on extraction; feasibility dependent on specific lead types used

## Patient selection

The choice between CSP and BiVP in patients with HFrEF and electrical dyssynchrony remains an active area of research, as it depends on multiple factors such as age, comorbidities, and anatomical considerations.

In patients with HFrEF and non-LBBB, the benefits of CRT with BiVP are significantly reduced. Observational studies suggest that CSP may offer advantages in these cases, showing greater electrical synchrony, reverse remodeling, improved cardiac function, and enhanced survival compared to BiVP, indicating that CSP may be a more effective approach for restoring ventricular synchrony in such patients (29).

Coronary artery disease (CAD) is also an important factor in selecting a pacing strategy. Patients with ischemic cardiomyopathy often show limited remodeling and less echocardiographic response following BiVP-CRT, likely due to extensive scar tissue. Additionally, CAD can increase the risk of rising capture thresholds during LBBAP implantation, possibly due to chronic ischemia, which affects myocardial tissue structure and cell excitability over time (30).

Ultrahigh-frequency ECG (UHF-ECG) has recently gained attention as a method for identifying electrical dyssynchrony through real-time instantaneous analyses and has been recently applied to evaluate ventricular activation in CSP. UHF-ECG showed that both CSP-CRT and BiVP-CRT reduce ventricular dyssynchrony significantly in patients with LBBB, with CSP-CRT being associated with shorter ventricular electrical delays and mean precordial depolarization times suggesting that CSP-CRT reduces ventricular dyssynchrony to a greater extent than BiVP-CRT (31). These findings suggest that UHF-ECG could be a valuable tool for developing a personalized resynchronization strategy, which helps guide the selection of the most effective pacing modality to achieve optimal ventricular synchrony (32).

Intraoperative assessment of interventricular conduction delay (IVCD) may aid in selecting the optimal CRT approach between BiVP and CSP, potentially improving the response rate to CRT. The IVCD can be evaluated intraoperatively by measuring RV-LV delay, and if RVs–LVs interval was less than 100 msec, then CSP might be the preferred option (33).

## Challenges and limitations of CSP

Although CSP is a promising tool to restore the electrical synchrony in patients with HFrEF, it carries some limitations including fluoroscopic evaluation of the anatomical position which may be imprecise, paced QRS morphology which may be affected by myocardial substrate and non-visualized conduction system potential. HBP itself has its own limitations including difficult His engagement in some cases, increasing capture threshold during follow-up and proximity to atrium resulting in oversensing and low sensing amplitude at the His bundle location. In contrast, LBBAP regions typically have higher sensing, lower capture thresholds, and similar-paced QRS durations (34).

Long-term lead performance, lead extractability, and impact on the tricuspid valve function represent the main concerns related to the CSP approach, and the data available so far are too scarce to draw conclusions on these matters. In particular, the impact of septal kinetics on lead durability and therefore on the evolution of the electrical parameters over time in the case of LBBAP is still not fully defined, although lumenless pacing leads could be less affected compared to stylet-driven pacing leads because of the smaller lead body and the high tensile strength (35).

Moreover, the availability of dedicated sheaths and different leads, including stylet-driven leads, has increased the probability of the LBBAP implant being successful. Stylet-driven leads offer higher LBBAP lead implantation success rates while shortening implant duration (36). However, there have been some concerns about early fracture of stylet-driven leads used with LBBAP due to higher mechanical stress on the distal part of the lead, making it more prone to fracture than in a conventional position (37, 38).

Distal conductor fractures in the interelectrode segment have been observed in stylet-driven leads, with up to 6.1% reported incidence rate of helix damage mainly attributed to entanglement with cardiac tissue, excessive angulation and preconditioning which could contribute to early fatigue fracture (39, 40). In contrast, lumenless leads usually feature a flexible, high-tensile, non-extensible inner conductor cable without a central lumen, making them less susceptible to conductor fractures than stylet-driven leads (40, 41).

On the other hand, Rangaswamy and his colleagues reported a case of late distal conductor fracture of the lumenless LBBAP lead occurring proximal to the ring electrode away from the lead-septum junction, possibly due to multiple attempts during the initial implantation process and suggesting minimizing the number of attempts by accurately identifying the target site before deploying the lead (40).

Data on lead extractability are currently limited to single-center experiences and case reports (42, 43). In a series involving 30 patients with chronically implanted lumenless His bundle leads (mean dwell time 25 ± 18 months), the extraction procedure had a success rate of over 95%, with no procedure-related complications reported. Most leads were extracted using simple traction, while mechanical extraction tools were necessary only in a few instances (43). For LBBP leads, existing case reports have demonstrated the feasibility of extraction procedures for lumenless leads implanted in the septal position for up to 3 years by gentle traction without complications (42, 44).

Preliminary observations in LBBP patients have shown worsening tricuspid valve regurgitation following the procedure, with some correlation noted between the implant site distance and the tricuspid valve annulus. These observations highlight the need to refine implant techniques to reduce lead interaction with the septal tricuspid leaflet and subvalvular apparatus. Additionally, incorporating imaging modalities, such as intracardiac echocardiography, to guide lead placement might help to optimize the outcomes as well (35, 45).

The CSP procedure can be challenging in patients with certain myocardial diseases, such as hypertrophic cardiomyopathy (HCM) with a thickened septum. Although a few case reports have demonstrated the feasibility of LBBP in HCM patients, technical difficulties might arise due to septal fibrosis, which may impact lead penetration and pacing thresholds. More evidence on long-term efficacy and safety is still required before CSP can be validated as a standard approach in such complex conditions (46, 47).

Additionally, CSP could be challenging in patients with congenital heart diseases, particularly after surgical correction/palliation of the primary defect or in the presence of prosthetic valves, surgical patches, or conduits as His bundle in these cases can be displaced from the expected position resulting in longer implant duration and higher radiation exposure. On the other hand, although the data on the safety and feasibility of CSP in CHD is scarce, CHD patients may derive the most benefit with physiological pacing considering their young age at the time of implant as well as the presence of structural heart disease, which has been associated with the risk of development of right ventricular pacing-induced cardiomyopathy. Furthermore, the procedure and fluoroscopy time of CSP in this population could be potentially reduced when guided by 3D electro-anatomical mapping (48–50).

Finally, device manufacturers have developed specialized implant tools for HBP and LBBAP to facilitate access to target pacing areas and enhance implant success rates. However, algorithms specifically designed for CRT in CSP settings are still lacking, particularly algorithms that can test and adjust pacing thresholds and sensitivity and adapt atrioventricular conduction intervals to enable fusion pacing in specific scenarios, such as selective LBBP, to prevent delayed right ventricular activation (51, 52).

## Gaps of evidence and ongoing trials

The rapid acceptance and evolution of CSP along with its safety has led to rapid growth of research in this field to assess safety, efficacy, and short- and long-term outcomes.

The ongoing Left vs. Left RCT (ClinicalTrials.gov identifier NCT05650658) is currently the largest clinical trial comparing CSP and BiVP in patients eligible for CRT. It aims to provide further clarity on the optimal CRT approach. This trial will enroll 2,136 patients with a minimum follow-up period of 3 years. Unlike earlier studies, the Left vs. Left RCT will be sufficiently powered to demonstrate superiority for the primary composite outcome of death and heart failure hospitalization. Currently, the study is in the feasibility phase and the full-scale study is projected to continue until 2029 (53).

Conduction System Pacing Versus Biventricular Pacing for Cardiac resYNChronization (CSP-SYNC) (ClinicalTrials.gov identifier NCT05155865) is another prospective, randomized trial that will also compare echocardiographic, electrocardiographic, and clinical outcomes of CSP versus conventional BiVP in HfrEF (LVEF ≤ 35%), sinus rhythm, and LBBB with a follow-up period for at least 6 months. The study will explore whether CSP is non-inferior to BiVP in echocardiographic, electrocardiographic, and clinical outcomes or not (54).

Similarly, Direct HIS/LBB Pacing as an Alternative to Biventricular Pacing in Patients with HFrEF and a Typical LBBB (HIS-alt_2) will investigate the feasibility of using direct HBP or LBBP as an alternative to BiVP in patients with symptomatic HFrEF and typical LBBB pattern (55).

The Left Bundle Cardiac Resynchronization Therapy trial (ClinicalTrials.gov identifier NCT05434962) will also enroll 176 patients with Class I or IIa indication for CRT according to current ESC or ACC/AHA/HRS guidelines and left bundle branch block and compare clinical and echocardiographic outcomes of LBBAP versus BiVP-CRT (56).

## Current guidelines recommendations for CSP in HFrEF and electrical dyssynchrony

The importance of conduction system pacing has been recognized in both European and American clinical guidelines. This concept was first mentioned in the 2018 American College of Cardiology/American Heart Association/Heart Rhythm Society (ACC/AHA/HRS) guidelines on bradycardia and cardiac conduction delay and the 2019 European Society of Cardiology (ESC) guidelines on supraventricular tachycardias (57, 58). However, there were no recommendations as first-line strategy or alternative strategy for CSP for patients with HFrEF and electrical dyssynchrony in these guidelines given the very limited data by that time.

Based on the promising data regarding the ventricular synchrony achieved by CSP, the 2021 ESC guidelines on cardiac pacing and CRT further expanded the use of HBP as rescue therapy in patients with unsuccessful coronary sinus lead implantation (Class IIa, Level of evidence B). However, no recommendations were made on LBBAP due to limited evidence at the time of the guidelines’ publication (2).

The most recent guidelines on cardiac physiologic pacing were published by the Heart Rhythm Society, Asia Pacific Heart Rhythm Society, and Latin American Heart Rhythm Society in 2023 recommending conduction system pacing as an initial strategy for patients with sinus rhythm, LVEF ≤ 35%, LBBB with QRSd ≥ 150 msec, and NYHA Class II–IV, (Class IIb, Level of evidence C), or as an alternative strategy to BiVP if effective CRT cannot be achieved with BiVP based on anatomical or functional criteria. (Class IIa, Level of evidence C). Additionally, these guidelines also expanded the role of cardiac physiologic pacing to include patients with LVEF ≤ 35%, sinus rhythm, a non-LBBB pattern with QRSd ≥ 150 msec, and NYHA Class II on GDMT (Class IIb, Level of evidence C) (59).

Table 3 provides a summary of the current guidelines recommendations for CSP in HFrEF and electrical dyssynchrony.

**Table 3:** Summary of current guidelines recommendations for CSP in HFrEF and electrical dyssynchrony.

Guideline	Recommendation for CSP	Level of evidence
2018 ACC/AHA/HRS Guidelines on Bradycardia and Conduction Delay	Recommends HBP as a pacing option for bradycardia and conduction delays but does not recommend it as a primary therapy for heart failure patients	-
2019 ESC Guidelines on Supraventricular Tachycardias	Recommends CSP as an alternative strategy for bradycardia pacing but not explicitly recommended for HFrEF and electrical dyssynchrony	-
2021 ESC Guidelines on Cardiac Pacing and CRT	Recommends HBP as a rescue therapy when coronary sinus lead implantation for CRT is unsuccessful	- Class IIa, Level B
2023 HRS/APHRS/LAHRS Guidelines on Physiologic Pacing	- Recommends CSP as an initial strategy for patients with sinus rhythm, LVEF ≤ 35%, LBBB with QRS ≥ 150 msec, and NYHA Class II–IV - Recommends CSP as an alternative to BiVP if CRT with BiVP is not effective based on anatomical/functional criteria	- Class IIb, Level C - Class IIa, Level C

## Conclusion

In conclusion, CSP is a safe and effective approach for reversing electrical dyssynchrony in patients with HFrEF who are candidates for CRT implantation. Due to its technical advantages and relatively shorter learning curve, LBBAP has the potential to become the standard approach for CRT in future clinical practice. However, large randomized controlled trials are necessary to compare the safety, efficacy, and both short- and long-term outcomes of various CSP techniques, particularly LBBAP versus BiVP.

## Glossary

ACC: American College of Cardiology
AF: atrial fibrillation
AHA: American Heart Association
BiVP: biventricular pacing
CAD: coronary artery disease
CRT: cardiac resynchronization therapy
CONSYST-CRT: Conduction System Pacing vs. Biventricular Resynchronization Therapy In Systolic Dysfunction and Wide QRS
CSP: conduction system pacing
ESC: European Society of Cardiology
GDMT: guideline-directed medical therapy
HBP: His bundle pacing
HF: heart failure
HFrEF: heart failure with reduced ejection fraction
HIS-alt_2: direct HIS/LBB pacing as an alternative to biventricular pacing in patients with HFrEF and a typical LBBB
HOT-CRT: His–Purkinje Conduction System Pacing Optimized Trial of Cardiac Resynchronization Therapy)
HRS: Heart Rhythm Society
IVCD: interventricular conduction delay
LBBB: left bundle branch block
LBBAP: left bundle branch area pacing
LBPP-RESYNC: Left Bundle Branch Pacing Versus Biventricular Pacing for Cardiac Resynchronization Therapy
LEVEL-AT: Left Ventricular Activation Time Shortening with Conduction System Pacing vs. Biventricular Resynchronization Therapy
LOT-CRT: Left Bundle Branch-Optimized Cardiac Resynchronization Therapy
LVEF: left ventricular ejection fraction
LVSP: left ventricular septal pacing
MW: myocardial work
NYHA: New York Heart Association
QRSd: QRS duration
RCT: randomized controlled trial
RVs–LVs: right ventricle sensed–left ventricle sensed interval
UHF-ECG: ultrahigh-frequency ECG
VF: ventricular fibrillation
VT: ventricular tachycardia.
